# Sphingosine synergizes with polymyxin antibiotics against Pseudomonas aeruginosa and Klebsiella pneumoniae

**DOI:** 10.1099/mic.0.001743

**Published:** 2026-08-04

**Authors:** Jacob R. Mackinder, Meghan Quinlan, Matthew J. Wargo

**Affiliations:** 1 Department of Microbiology and Molecular Genetics, Larner College of Medicine, University of Vermont, Burlington, USA; 2 Cellular, Molecular, and Biomedical Sciences Graduate Program, University of Vermont, Burlington, USA

**Keywords:** antibiotic synergy, antimicrobial resistance, sphingosine, polymyxin

## Abstract

Antimicrobial resistance is an increasing threat to global health. However, there is a limited set of antibiotics that are effective against drug-resistant Gram-negative bacteria like *Pseudomonas aeruginosa*. One strategy to enhance the efficacy and longevity of existing antibiotics is by combining them with non-traditional antimicrobial adjuvants. Here, we examined if the host-derived antimicrobial lipid sphingosine could enhance the efficacy of a panel of antibiotics against *P. aeruginosa in vitro*. We found that sphingosine displayed strong synergy with the polymyxin antibiotics, polymyxin B and colistin, to inhibit growth of and kill *P. aeruginosa* but did not significantly alter the efficacy of other tested antibiotic classes. The addition of sphingosine reduced the MIC of polymyxin B and colistin from 0.5 to 0.031 µg ml^−1^ and 8 to 0.5 µg ml^−1^, respectively. This combination of sphingosine and polymyxin B synergized to inhibit the growth and survival of *Klebsiella pneumoniae*, as well as clinically isolated of *P. aeruginosa*. In addition to sphingosine, we found that the sphingoid bases sphinganine (dihydrosphingosine) and phytosphingosine also enhanced the activity of polymyxins. Overall, these findings demonstrate that sphingosine is a potent adjuvant for polymyxins and that the sphingosine–polymyxin combination is capable of killing *P. aeruginosa* and *K. pneumoniae* while using relatively low concentrations of polymyxin. This study may help in the development of new antimicrobial therapies for the treatment of Gram-negative bacterial infections.

## Introduction

Antibiotic resistance is a major problem with the number of antibiotic-resistant infections increasing [1]. Many antibiotic-resistant infections can be attributed to a specific group of bacteria referred to as the ‘ESKAPE’ pathogens, which include *Enterococcus faecium*, *Staphylococcus aureus*, *Klebsiella pneumoniae*, *Acinetobacter baumannii*, *Pseudomonas aeruginosa* and other members of the *Enterobacterales* group [2, 3]. ESKAPE pathogens have been designated ‘priority status’ by the World Health Organization (WHO) with the goal to increase research, discovery and development of new antibiotics to combat these pathogens.

*P. aeruginosa* is a Gram-negative opportunistic bacterial pathogen capable of infecting numerous body sites including the lungs, bloodstream, urinary tract, burns and surgical wounds [4–7]. Its success as an opportunist is partially due to its ability to rapidly adapt to environmental cues and stressors, including antibiotic exposure [8–11]. *P. aeruginosa* has a variety of intrinsic and acquired antimicrobial resistance mechanisms, which include biofilm formation, reduced outer membrane permeability, efflux systems and drug-inactivating enzymes [10–14]. Multidrug-resistant (MDR) strains of *P. aeruginosa* are often associated with hospital- and ventilator-acquired pneumonia, bacteraemia and chronic lung infections in individuals with cystic fibrosis (CF) and chronic obstructive pulmonary disease [4–6, 10, 15–17]. From 2015 to 2017, the National Healthcare Safety Network found that 26% of *P. aeruginosa* isolates from intensive care unit patients were resistant to carbapenems, 26.5% were resistant to extended-spectrum cephalosporins, 27.1% were resistant to fluoroquinolones and 18.6% were resistant to three or more antibiotics [17]. Although most *P. aeruginosa* infections can be treated with currently available antibiotics, the continued increase of antibiotic resistance rates combined with limited development of new antimicrobials is a dire situation [18]. Therefore, there is an urgent need to develop novel antimicrobial strategies, one of which is through the combination of existing antibiotics with non-traditional adjuvants.

Antimicrobial combinations that repurpose existing antibiotics offer a sustainable and cost-effective alternative to the discovery and development of novel antibiotics. Combinatorial therapies can result in improved outcomes over monotherapy when the primary drug alone has a high likelihood of inducing resistance (e.g. fosfomycin), when the pathogen has a high rate of developing resistance (e.g*. P. aeruginosa*), or when there are limited treatments for highly resistant infections in vulnerable patients [10, 11, 19, 20]. The best studied combinatorial therapies for drug-resistant infections are *β*-lactam antibiotics paired with *β*-lactamase inhibitors; however, host-derived antimicrobial factors have also been explored [21–25]. Sphingolipids are a class of lipids primarily found in eukaryotes that have a variety of biological functions, including immune signalling and antimicrobial activity [25–27]. The sphingoid base sphingosine, the core moiety of most sphingolipids, has potent antimicrobial properties, capable of inhibiting growth and killing both Gram-negative and Gram-positive bacteria [26, 28–30]. In the case of Gram-negative bacteria, sphingosine destabilizes the outer membrane by binding to cardiolipin, resulting in increased cardiolipin turnover and degradation with eventual leakage of ATP and loss of metabolic activity [31, 32]. The anti-infectious role of sphingosine in epithelial tissues has been studied primarily in the contexts of atopic dermatitis and CF, where the reduction of local sphingosine concentrations in the stratum corneum and airway lumen, respectively, leaves these tissues more susceptible to infection [33–36]. Restoring the sphingolipid homeostasis in CF mice through inhalation of aerosolized sphingosine or recombinant acid ceramidase, which releases sphingosine from accumulated ceramide, reduces infection and improves survival outcomes [35–37]. The anti-infectious role of sphingosine led us to test whether this host compound would be able to synergize with currently available antibiotics to inhibit growth of *P. aeruginosa*.

## Methods

### Strains and growth conditions

*P. aeruginosa* (PA14 and PAO1) and *K. pneumoniae* (MGH78578 and KPPR1) strains were maintained at 37 °C on lysogeny broth Lennox formulation. In preparation for assays, *P. aeruginosa* was grown overnight in modified MOPS medium [38, 39] supplemented with 20 mM pyruvate and 5 mM glucose, whereas *K. pneumoniae* was grown overnight in modified MOPS medium supplemented with 20 mM lactate and 5 mM glucose.

The clinical isolates used here were MJ1216, JR398 and JR399. MJ1216 is a recent *P. aeruginosa* osteomyelitis isolate that was resistant to all single antibiotics tested in the local hospital laboratory, and testing by the Mayo Clinic during patient treatment showed resistance to ceftazidime/avibactam, ceftolozane/tazobactam and imipenem–relebactam, with intermediate susceptibility (~2 µg ml^−1^) to colistin and susceptibility only to cefiderocol. JR398 is an isolate from CF sputum and JR399 is a urine isolate; both are resistant to gentamicin, kanamycin, carbenicillin and trimethoprim and susceptible to tobramycin, ciprofloxacin and ceftazidime based on testing in our lab. These latter two strains were gifts from Barbara Kazmierczak and Thomas Murray.

### Chemicals, antibiotics and notes on sphingosine stability and solubility

All media and standard chemicals were purchased from ThermoFisher (Waltham, MA) or Sigma-Aldrich (St. Louis, MO). Gentamicin, tobramycin sulphate, aztreonam, ceftazidime, ciprofloxacin and vancomycin hydrochloride were purchased from ThermoFisher. Polymyxin B sulphate and colistin sulphate were both obtained from MP Biomedicals (Irvine, CA). Antibiotics were stored at −20 °C as single-use aliquots to avoid multiple freeze-thaw cycles. The sphingoid bases sphingosine, sphinganine and phytosphingosine were purchased from Cayman Chemical (Ann Arbor, MI) and dissolved in 95% ethanol to make 50 mM stocks. Sphingoid bases were stored as aliquots at −20 °C to avoid multiple freeze-thaw cycles, which impact the antibacterial activity of the lipids [9, 40, 41]. Sphingoid bases were administered to the multi-well plates in ethanol and allowed to air dry to remove solvent prior to media addition.

### Combination antimicrobial susceptibility assays

Antimicrobial susceptibility assays to determine MICs were conducted by the broth microdilution method as recommended by the CLSI guidelines [42] with the exception that MOPS media was used instead of Mueller–Hinton broth (MHB) (see Discussion). Serial twofold dilutions of antibiotics were prepared in modified MOPS medium supplemented with 20 mM of pyruvate or lactate for growth of *P. aeruginosa* and *K. pneumoniae*, respectively, and added to the 96-well plates containing the dried sphingoid bases. Overnight cultures were harvested by centrifugation, washed in MOPS media and resuspended in MOPS with 20 mM pyruvate for *P. aeruginosa* or 20 mM lactate for *K. pneumoniae* at an OD_600_ of 0.01 or 0.001, respectively. Adjusted cultures were then added to the 96-well plates containing antibiotic and sphingoid bases at a 1:10 (v:v) ratio for a starting OD_600_ of 0.001 (1×10^6^ c.f.u. ml^−1^) or 0.0001 (1×10^5^ c.f.u. ml^−1^) for *P. aeruginosa* and *K. pneumoniae*, respectively. The difference in initial OD_600_ is to correct for the faster growth rate of *K. pneumoniae* compared to *P. aeruginosa* [43] in these media conditions. To allow for equal gas exchange in each well, 96-well plates were covered with sterile microporous sealing film from USA Scientific (Ocala, FL). Cultures were grown for 18 h at 37 °C with horizontal shaking at 170 r.p.m. The OD_600_ was measured using a BioTek Synergy H1 plate reader at 0 and 18 h to assess growth. To assess the change in growth after 18 h, the OD_600_ at 0 h was subtracted from the 18 h reading, and the difference was then divided by an untreated control. MICs were determined by the lowest concentration of antibiotic with or without sphingoid base that inhibited ≥90% of growth, relative to an untreated control.

### Checkerboard assays to determine fractional inhibitory concentrations

Similar to MIC assays, for checkerboard assays, sphingoid bases were diluted separately to 5 mM, 500 µM and 250 µM stocks and applied directly to the 96-well plates used for assays to create a range of twofold dilutions from 200 to 3.125 µM. After the ethanol vehicle had evaporated, twofold serial dilutions of antibiotic in MOPS with 20 mM pyruvate or 20 mM lactate were added to plates containing the dried sphingoid bases. Like the MIC assays, washed and adjusted cultures were added to assay plates at a starting OD_600_ of 0.001 and 0.0001 for *P. aeruginosa* and *K. pneumoniae*, respectively. Assay plates were sealed with breathable microporous film, and cultures were grown for 18 h at 37 °C with horizontal shaking at 170 r.p.m. The OD_600_ was measured using a BioTek Synergy H1 plate reader at 0 and 18 h to assess growth. To assess the change in growth after 18 h, the OD_600_ at 0 h was subtracted from the 18 h reading, and the difference was then divided by an untreated control. MICs were determined by the lowest concentration of antibiotic with or without sphingoid base that inhibited ≥90% of growth, relative to an untreated control. MIC values were used to calculate the fractional inhibitory concentration index (FICI) of the combinations (Equation 1). The combined effects of the antimicrobials were characterized as synergistic, additive, unrelated and antagonistic when the FICI was ≤0.5, 0.5–1.0, 1–2 and >2, respectively [44].


(1) $$FICI={FIC}_{A}+{FIC}_{B}=\frac{C_{A}}{MIC_{A}}+\frac{C_{B}}{MIC_{B}}$$

In Equation 1, the FICI represents the sum of the fractional inhibitory concentrations (FICs) (FIC_A_ and FIC_B_) of each drug tested, which are determined for each drug by dividing the MIC of each drug when used in combination (*C*_A_ and *C*_B_) by the MIC of each drug when used alone (MIC_A_ and MIC_B_).

### Minimum bactericidal concentration assays

To observe the bactericidal effects of polymyxins and sphingosine, minimum bactericidal concentration (MBC) of polymyxin B and sphingosine combinations were determined after checkerboard assays. After 18 h, 10 µl was removed from each well of the checkerboard and spotted onto *Pseudomonas* isolation agar or Reasoner’s 2A agar for growth of *P. aeruginosa* and *K. pneumoniae*, respectively. MBCs were determined by the lowest concentration of antimicrobials alone or in combination where no colonies were observed. The limit of detection was set at 100 c.f.u. ml^−1^.

### Data acquisition

All data gathered is from three independent experiments, each with technical duplicates. Results were presented as mean±sem. Data collected using a BioTek Synergy H1 were exported to Microsoft Excel, and all normalization and fold change calculations were done in Excel. GraphPad Prism was used for visualization.

## Results

### Screen of antibiotics for altered activity with sphingosine

To test if sphingosine could be used as an antimicrobial adjuvant, we measured *P. aeruginosa* growth in a panel of CF-relevant antibiotics, covering five antibiotic classes: aminoglycosides (gentamicin and tobramycin), *β*-lactams (aztreonam and ceftazidime), cyclic polypeptides (polymyxin B and colistin), a fluoroquinolone (ciprofloxacin) and a glycopeptide (vancomycin) (Table 1). *P. aeruginosa* cultures were grown in minimal media containing serial dilutions (1:1, v:v) of antibiotic with or without 20, 70 or 200 µM sphingosine. These sphingosine concentrations were selected because they do not inhibit wild-type *P. aeruginosa* growth [40] and are found in physiologically relevant environments of *P. aeruginosa* infection: airway surface liquid (20 µM), nasal mucosa (70 µM) and the stratum corneum of the skin (200 µM) [33, 45–51].

**Table 1.** Mean MICs of antibiotics screened alone or with 200 µM sphingosine

Class	Antibiotic	Target	PA14	PA14	PAO1*	PAO1*
			MIC (µg ml^−1^)	MIC_combination_ (µg ml^−1^)	MIC (µg ml^−1^)	MIC_combination_ (µg ml^−1^)
Aminoglycoside	Gentamicin	Ribosome	2	2	2	4
	Tobramycin	Ribosome	1	2	1	2
*β*-Lactam	Aztreonam	Peptidoglycan	1	1	–	–
	Ceftazidime	Peptidoglycan	4	4	–	–
Fluoroquinolone	Ciprofloxacin	Topoisomerase	1	2	–	–
Glycopeptide	Vancomycin	Peptidoglycan	>512	>512	–	–
Polymyxins	Polymyxin B	Outer membrane	0.5	0.03125	0.5	0.125
	Colistin	Outer membrane	8	0.5	8	2

*PAO1 was not tested against *β*-lactam, fluoroquinolone or glycopeptide antibiotics. –, Not calculated.

**Fig. 1.**
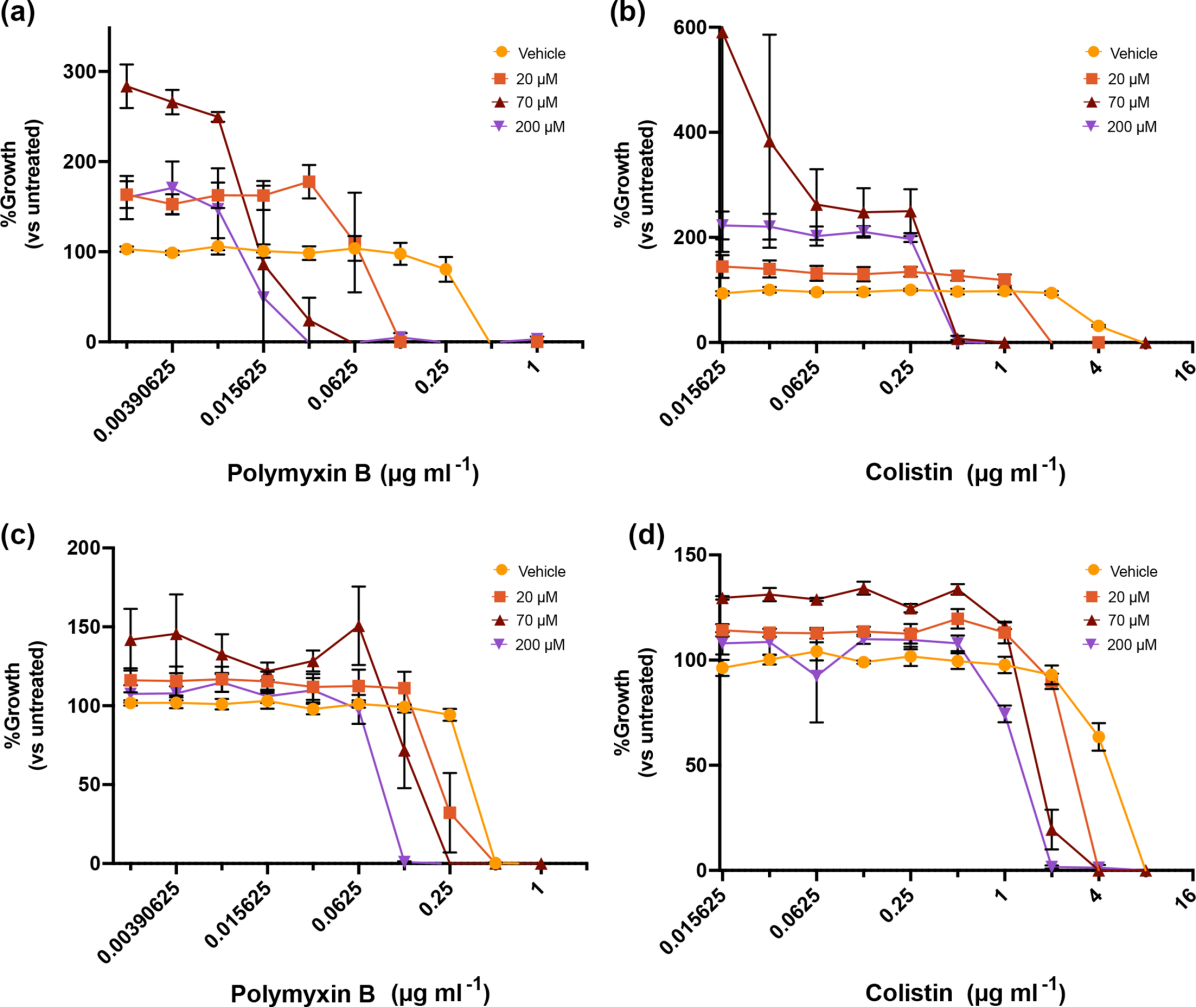
Sphingosine reduces MIC of polymyxin antibiotics. Dose-effect curves of PA14 in increasing concentrations of polymyxin B (**a**) or colistin (**b**) alone or with 20, 70 and 200 µM sphingosine. Dose-effect curves of PAO1 in increasing concentrations of polymyxin B (**c**) or colistin (**d**) alone or with 20 70 and 200 µM sphingosine. Data points denote means summarizing three independent experiments, and error bars represent the sem.

Regardless of the concentration, sphingosine did not alter the MICs of aztreonam, ceftazidime or vancomycin (Table 1**,** Figs S1–S5, available in the online Supplementary Material). The presence of sphingosine (20, 70 and 200 µM) had a small effect on the MIC of aminoglycosides, shifting it from 1 to 2 µg ml^−1^ or 4 µg ml^−1^ for tobramycin and gentamicin, respectively (Figs S1 and S2). Likewise, 200 µM of sphingosine moved the MIC of ciprofloxacin from 1 to 2 µg ml^−1^ (Fig. S3). Conversely, the addition of sphingosine to the two polymyxin antibiotics, polymyxin B and colistin (polymyxin E), substantially reduced the MIC of both drugs. The MIC of polymyxin B dropped from 0.5 µg ml^−1^ alone to 0.125 and 0.031 µg ml^−1^ with the addition of 20 µM and either 70 or 200 µM sphingosine, respectively (Fig. 1a, Table 1); thus, either 70 or 200 µM sphingosine reduced the polymyxin B MIC 16-fold. The addition of 20 µM sphingosine reduced the MIC of colistin from 8 to 2 µg ml^−1^, while the addition of either 70 or 200 µM sphingosine reduced the MIC of colistin to 0.5 µg ml^−1^ (Fig. 1b, Table 1), the latter representing approximately a 16-fold reduction in colistin concentration. In addition to PA14, 200 µM sphingosine enhanced polymyxin antibiotics against PAO1 with a shift in the MIC of polymyxin B from 0.5 to 0.125 µg ml^−1^ and the MIC of colistin dropped from 8 to 2 µg ml^−1^ (Fig. 1c, d, Table 1). Because PA14 displayed the strongest level of synergy, it was used for subsequent experiments.

### Sphingosine and polymyxin antibiotics synergize to inhibit *P. aeruginosa* growth

**Fig. 2.**
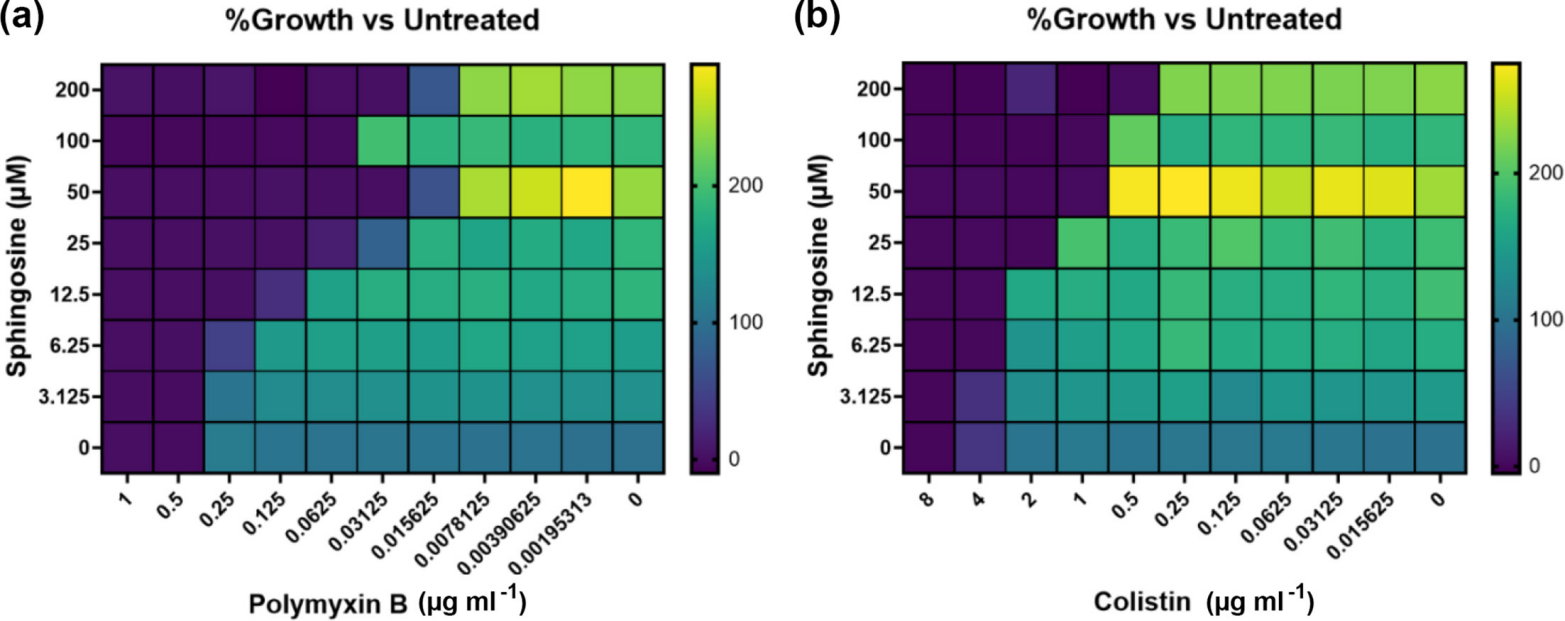
Checkerboard assays of *P. aeruginosa* growth with combinations of sphingosine and polymyxin antibiotics. Heatmaps representing percent PA14 growth in polymyxin B (**a**) and colistin (**b**) with a series of sphingosine concentrations. Presented as the mean percent growth relative to the untreated control from three independent experiments.

To determine if the enhanced antibiotic activity of polymyxins and sphingosine represented antibiotic synergy, we conducted checkerboard assays with serial dilutions of both compounds. Checkerboard assays were used to calculate the FICI of the combinations (**Equation 1**). For categorization of FIC values, we used the definitions of ≤0.5 as synergistic, 0.5–1.0 as additive, 1–2 as unrelated and >2 as antagonistic [44]. Sphingosine enhanced the efficacy of both polymyxin antibiotics at multiple concentrations. Synergy between sphingosine and polymyxin B was observed when 50 µM sphingosine was combined with 0.031 µg ml^−1^ polymyxin B and when 25 µM sphingosine was used with 0.125 µg ml^−1^ polymyxin B, resulting in FICIs of 0.26 and 0.35, respectively (Fig. 2a, Table 2). Colistin combined with sphingosine resulted in an FICI ranging from 0.325 to 0.35 when using 50 µM sphingosine and 1 µg ml^−1^ colistin or 25 µM sphingosine with 2 µg ml^−1^ colistin, respectively (Fig. 2b, Table 2).

**Table 2.** MICs and FICIs of sphingoid bases and polymyxin antibiotics

	Sphingoid base			Antibiotic			FICI combination*	Interpretation†
Strain	Compound	MIC (µM)	MIC_combination_ (µM)	Compound	MIC (µg ml^−1^)	MIC_combination_ (µg ml^−1^)		
*P. aeruginosa* PA14	Sphingosine	>250	200	Polymyxin B	0.5	0.03125	0.8625	Additive
			100			0.0625	0.525	Additive
			50			0.03125	0.2625	Synergistic
			25			0.125	0.35	Synergistic
			12.5			0.25	0.55	Additive
			200	Colistin	8	0.5	0.8625	Additive
			100			1	0.525	Additive
			50			1	0.33	Synergistic
			25			2	0.35	Synergistic
			12.5			4	0.55	Additive
			6.25			4	0.525	Additive
	Sphinganine	>250	50	Polymyxin B	0.5	0.25	0.7	Additive
	Phytosphingosine	50	25	Polymyxin B	0.5	0.125	0.75	Additive
			12.5			0.25	0.75	Additive
*K. pneumoniae* MGH78578	Sphingosine	>250	200	Polymyxin B	0.5	0.0078125	0.815625	Additive
			100			0.015625	0.43125	Synergistic
			50			0.0078125	0.215625	Synergistic
			25			0.03125	0.1625	Synergistic
			12.5			0.0625	0.175	Synergistic
			6.25			0.25	0.525	Additive
			3.125			0.25	0.5125	Additive
*K. pneumoniae* KPPR1	Sphingosine	>250	200	Polymyxin B	0.5	0.015625	0.83125	Additive
			100			0.125	0.65	Additive
			50			0.0625	0.325	Synergistic
			6.25			1	2.025	Antagonistic
			3.125			1	2.0125	Antagonistic

*FICI ≤0.5, >0.5; ≤1, >1; ≤2; and >2 represent synergistic, additive, unrelated and antagonistic effects, respectively [44].

†Unrelated interactions were not included for brevity.

### Other sphingoid bases enhance the efficacy of polymyxin B against *P. aeruginosa*

**Fig. 3.**
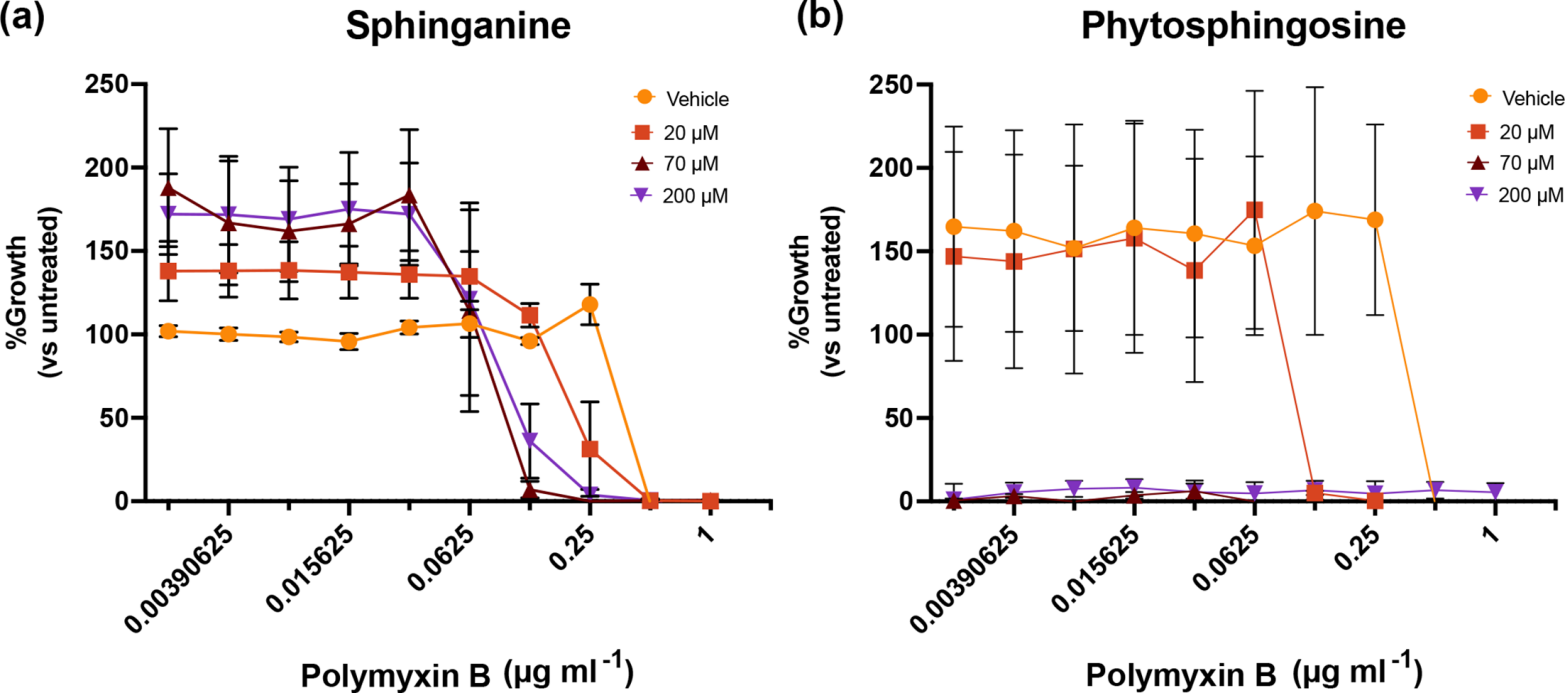
Sphingoid bases reduce polymyxin B MIC. Dose-effect curves of PA14 in increasing concentrations of polymyxin B in combination with sphinganine (**a**) and phytosphingosine (**b**). Data points denote means summarizing three independent experiments, and error bars represent the sem.

In addition to sphingosine, the sphingoid bases sphinganine (dihydrosphingosine) and phytosphingosine display antimicrobial activity [26–30]. Therefore, like sphingosine, we conducted the MIC assays using these two lipids to determine whether they can enhance polymyxin B efficacy. Both sphinganine and phytosphingosine enhanced the antibacterial effects of polymyxin B. The MIC of polymyxin B did not change with 20 µM sphinganine, but in combination with 70 and 200 µM, the MIC of polymyxin B dropped from 0.5 to 0.25 µg ml^−1^ (Fig. 3a). The addition of 20 µM phytosphingosine to polymyxin B reduced the MIC from 0.5 to 0.125 µg ml^−1^ (Fig. 3b). *P. aeruginosa* did not grow at any concentration of antibiotic when 70 or 200 µM phytosphingosine was present, suggesting that the MIC of phytosphingosine was between 20 and 70 µM.

**Fig. 4.**
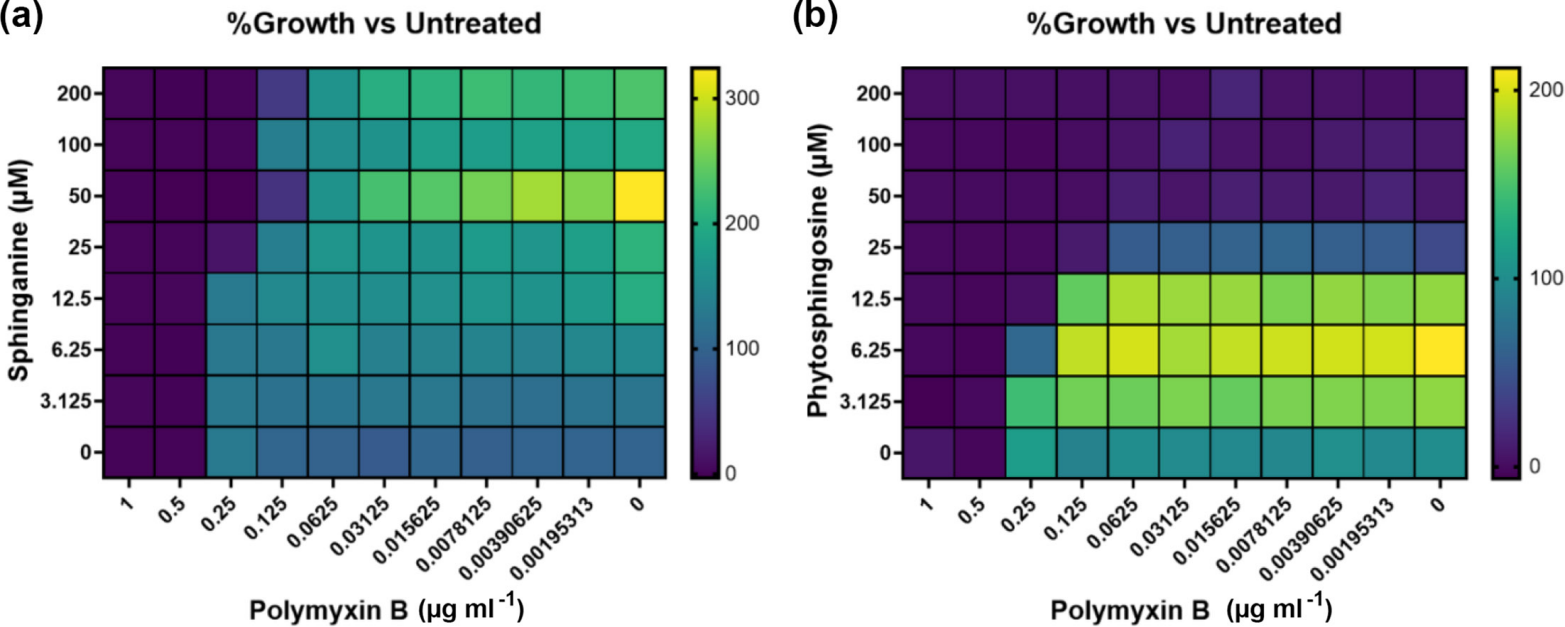
Checkerboard assays of *P. aeruginosa* growth with combinations of sphingoid bases and polymyxin B. Heatmaps representing percent PA14 growth in different combinations of polymyxin B with sphinganine (**a**) and phytosphingosine (**b**). Presented as the mean percent growth relative to the untreated control from three independent experiments.

**Fig. 5.**
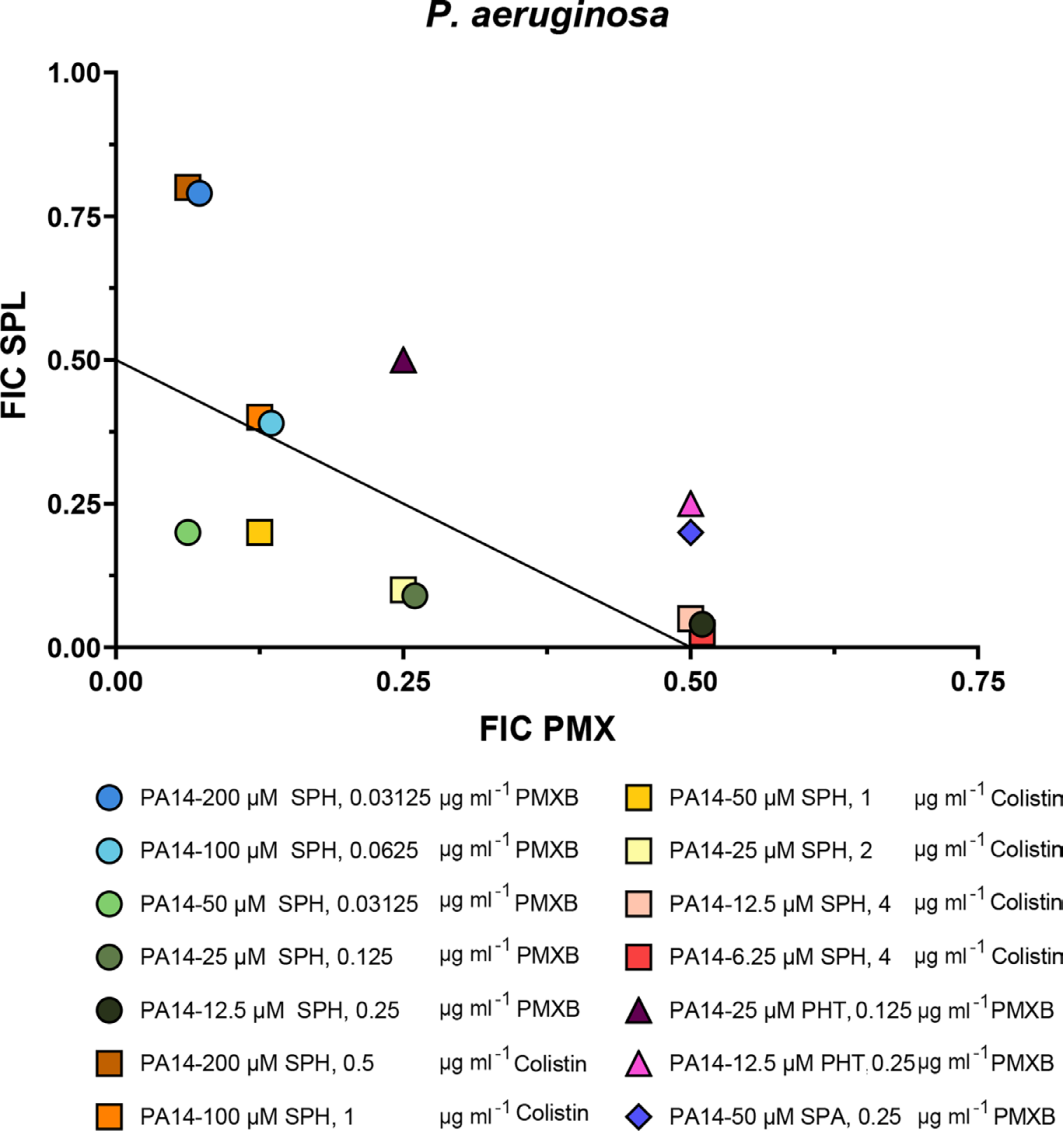
Isobologram of FICI values determined from PA14 checkerboard assays. FIC of sphingoid bases (FIC SPL) were plotted against the FIC of polymyxin antibiotics (FIC PMX). Combinations of sphingoid bases and polymyxins that inhibited bacterial growth are presented as isobolograms where the FICs of sphingoid bases (FIC SPL) were plotted against the FIC of polymyxin antibiotics (FIC PMX). The slope of the line represents the cutoff for synergistic effects between the two compounds (FICI <0.5); therefore, points plotted below the line represent synergistic effects, while points at or above the line represent additive (0.5<FICI≤1), unrelated (1<FICI≤2) and antagonistic effects (FICI >2) [44].

Because we observed enhanced antibacterial activity of polymyxin B in combination with sphinganine and phytosphingosine, we conducted checkerboard assays to determine if this enhancement could be defined as synergy with polymyxin B. The combination of 50 µM sphinganine and 0.25 µg ml^−1^ polymyxin B had an FICI of 0.70, representing an additive effect (Fig. 4a, Table 2). The checkerboards containing mixtures of polymyxin B and phytosphingosine revealed that 50 µM phytosphingosine alone was sufficient to fully inhibit *P. aeruginosa* growth. The addition of 12.5 µM phytosphingosine to 0.25 µg ml^−1^ polymyxin B displayed an additive effect against *P. aeruginosa* growth with an FICI of 0.75; the same FICI was produced from the combination of 25 µM phytosphingosine and 0.125 µg ml^−1^ polymyxin B (Fig. 4b, Table 2). FIC values of all sphingoid bases with polymyxins were plotted on isobolograms to illustrate their combined effects on *P. aeruginosa* growth (Fig. 5).

### Sphingosine synergizes with polymyxin B to inhibit growth of clinical *P. aeruginosa* strains

**Fig. 6.**
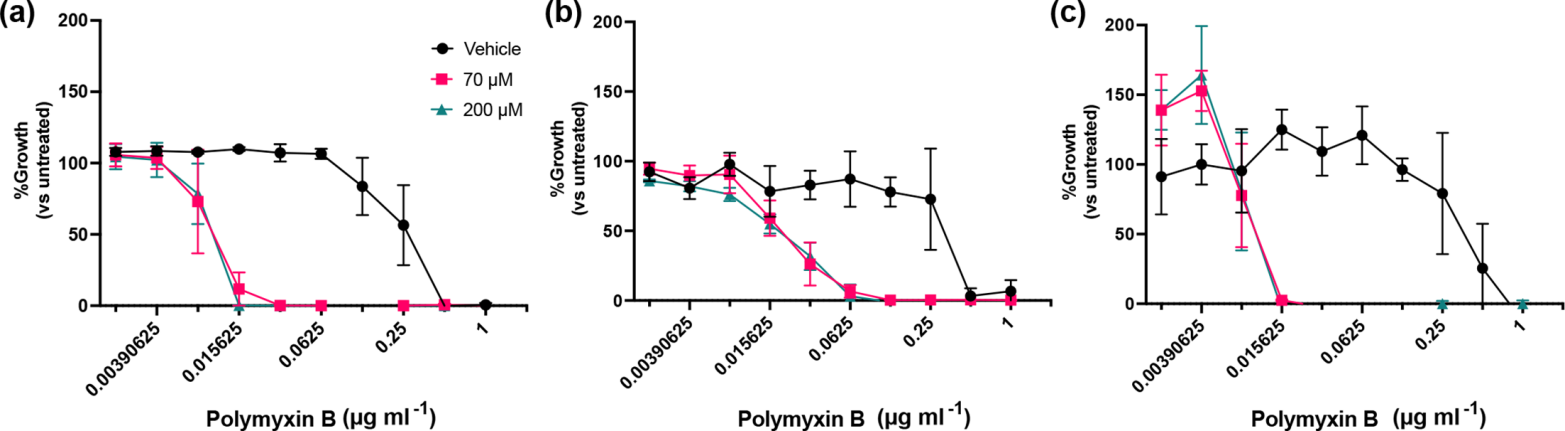
Sphingosine increases effectiveness of polymyxin antibiotics in clinical *P. aeruginosa* isolates. Dose-effect curves of three clinical *P. aeruginosa* isolates in increasing concentrations of polymyxin B alone or with 70 or 200 µM sphingosine. (**a**) MDR *P. aeruginosa* MJ1216 from osteomyelitis. (**b**) *P. aeruginosa* isolate JR398 from sputum. (**c**) *P. aeruginosa* isolate JR399 from urine. Data points denote means summarizing three independent experiments, and error bars represent the sem.

While PAO1 and PA14 are both derived from human infection, they are generally considered laboratory strains. To assess the efficacy on *P. aeruginosa* clinical isolates, we tested sphingosine and polymyxin B dose-effect on clinical isolates from urine, sputum and osteomyelitis. The osteomyelitis isolate, MJ1216, was isolated in 2025 and is considered MDR based on clinical treatment and genome sequence (GenBank JBWBCO000000000.1). All three of these clinical isolates of *P. aeruginosa* were made much more susceptible to polymyxin B by co-treatment with sphingosine (Fig. 6).

### Sphingosine synergizes with polymyxin B to inhibit growth of *K. pneumoniae*

**Fig. 7.**
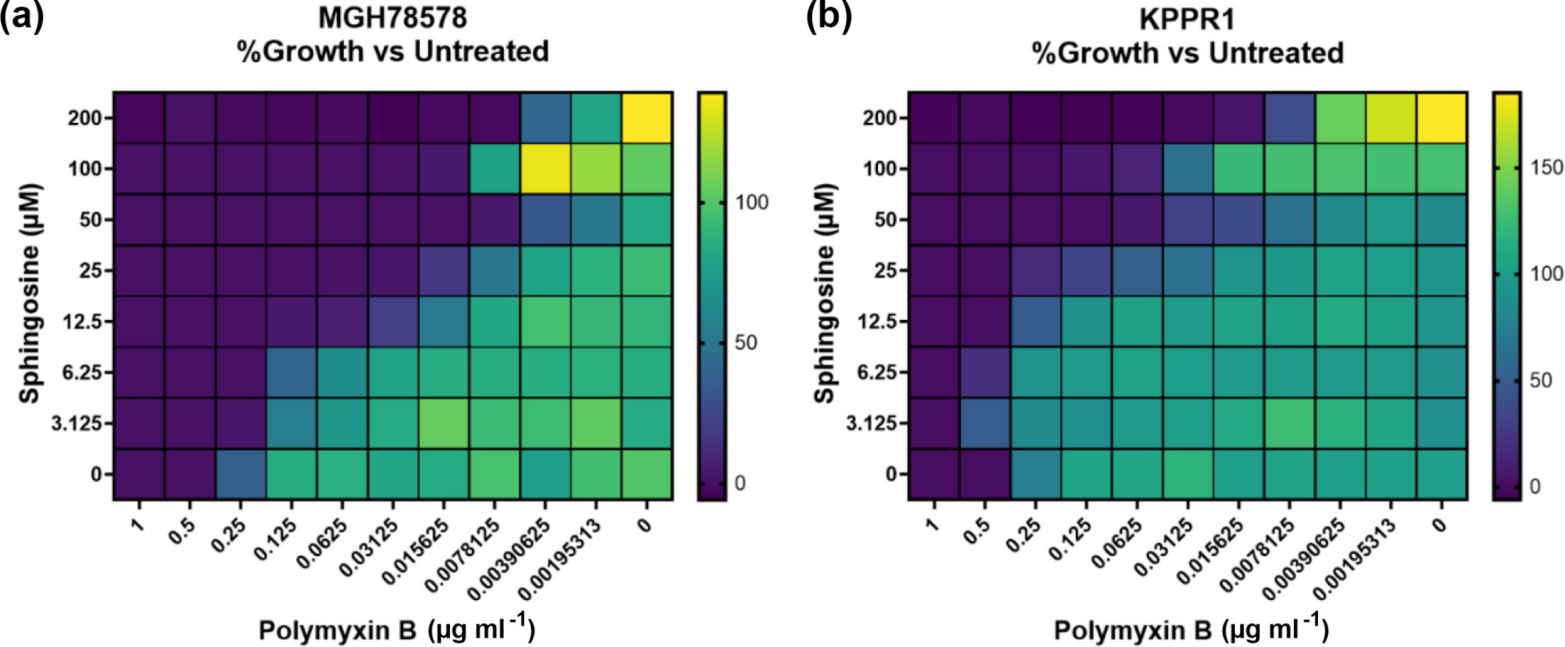
Checkerboard assays of *K. pneumoniae* growth with combinations of sphingosine and polymyxin antibiotics. Heatmaps representing percent growth of *K. pneumoniae* strains MGH78578 (**a**) and KPPR1 (**b**) in different combinations of polymyxin B and sphingosine. Presented as the mean percent growth relative to the untreated control from three independent experiments.

**Fig. 8.**
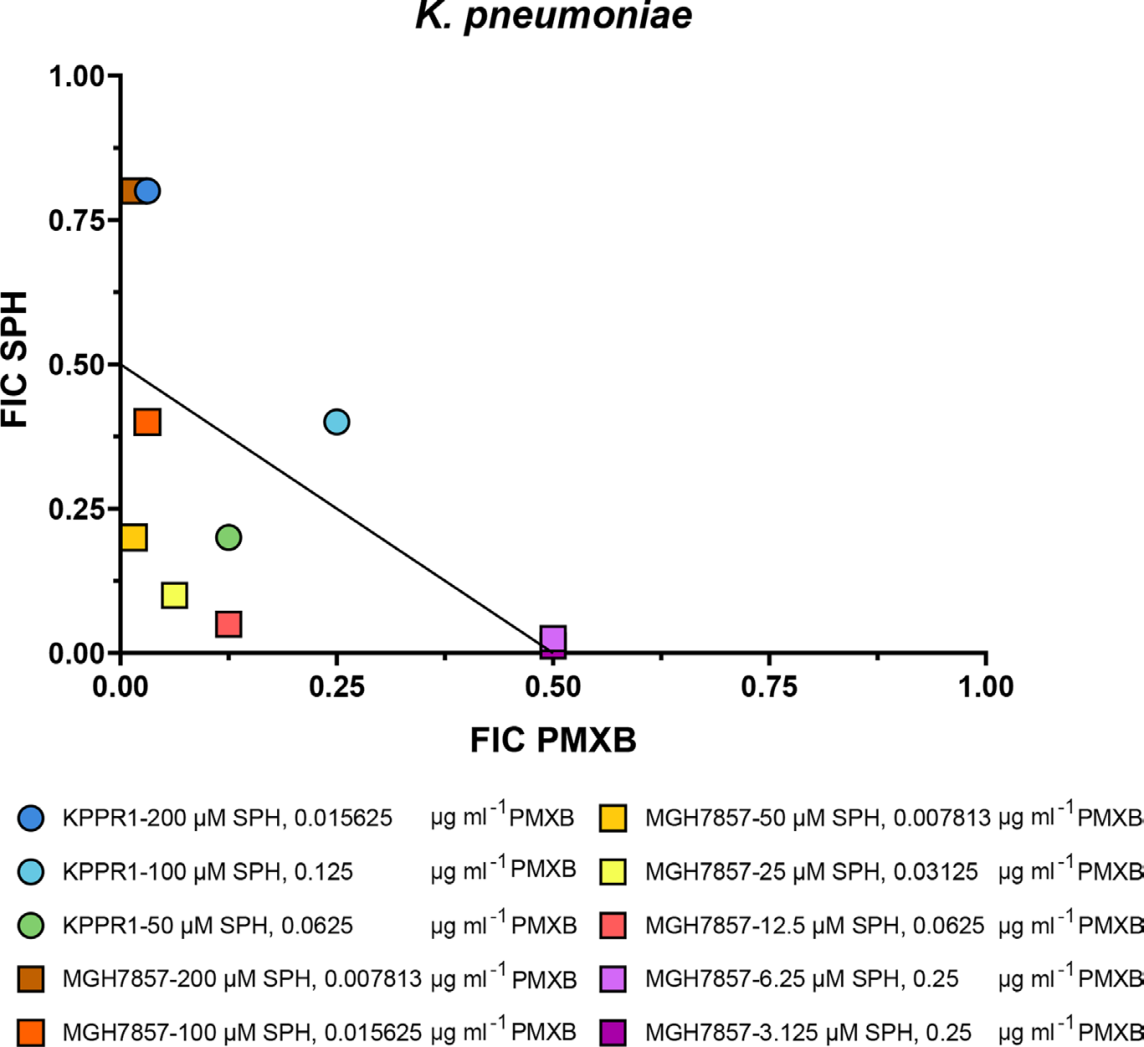
Isobologram of FICI values determined from *K. pneumoniae* checkerboard assays. FIC of sphingosine (FIC SPH) were plotted against the FIC of polymyxin B (FIC PMXB). Combinations of sphingoid bases and polymyxins that inhibited bacterial growth are presented as isobolograms where the FICs of sphingoid bases (FIC SPL) were plotted against the FIC of polymyxin antibiotics (FIC PMX). The slope of the line represents the cutoff for synergistic effects between the two compounds (FICI <0.5); therefore, points plotted below the line represent synergistic effects, while points at or above the line represent additive (0.5<FICI≤1), unrelated (1<FICI≤2) and antagonistic effects (FICI >2) [44]. Antagonistic effects highlighted in Fig. 7 are not shown on this plot for clarity.

In addition to *P. aeruginosa*, we were interested in the combined activity of sphingosine and polymyxin B on another antibiotic-resistant opportunistic pathogen, *K. pneumoniae*. Specifically, we focused on combinations of sphingosine and polymyxin B against two *K. pneumoniae* strains: MGH78578 and KPPR1. Both *K. pneumoniae* strains were inhibited by 0.5 µg ml^−1^ of polymyxin B alone, but not with sphingosine alone. Growth of MGH78578 was inhibited at multiple combinations of sphingosine and polymyxin B (Fig. 7a), resulting in an FICI range of 0.16–0.43 (Fig. 8, Table 2). The combination of 25 µM sphingosine and 0.031 µg ml^−1^ polymyxin B had the greatest synergistic interaction with an FICI of 0.1625. When KPPR1 was challenged with sphingosine and polymyxin B, 50 µM sphingosine displayed synergy with 0.063 µg ml^−1^ polymyxin B with an FICI of 0.325 (Figs 7b and 8, Table 2). Interestingly, the combination of 1 µg ml^−1^ polymyxin B and either 6.25 µM or 3.125 µM sphingosine resulted in a slight antagonistic interaction with FICI of 2.025 and 2.0125, respectively (Fig. 8, Table 2).

### Sphingosine and polymyxin combine to enhance killing of *P. aeruginosa* and *K. pneumoniae*

In addition to growth inhibition, we wanted to observe what concentration of polymyxin B and sphingosine resulted in killing of *P. aeruginosa* and *K. pneumoniae*. In our system, sphingosine alone has not displayed bactericidal activity against *P. aeruginosa* and *K*. pneumoniae, while 0.5 µg ml^−1^ of polymyxin B is sufficient to kill either bacterium. Against *P. aeruginosa,* the presence of 50 µM sphingosine reduced the MBC of polymyxin B to 0.031 µg ml^−1^ (Table 3). We observed that the lowest concentration of polymyxin B needed to kill either strain of *K. pneumoniae* was with the addition of 200 µM sphingosine. With 200 µM sphingosine, the MBC of polymyxin B for MGH78578 and KPPR1 was 0.063 and 0.047 µg ml^−1^, respectively (Table 3).

**Table 3.** Median MBCs of polymyxin B in combination with sphingosine

	MBC of polymyxin B (µg ml^−1^)		
Sphingosine (µM)	*P. aeruginosa* PA14	*K. pneumoniae* MGH78578	*K. pneumoniae* KPPR1
**200**	0.0625	0.0625	0.046875
**100**	0.125	0.25	0.1875
**50**	0.03125	0.15625	0.15625
**25**	0.09375	0.25	0.5
**12.5**	0.25	0.5	0.75
**6.25**	0.5	0.75	1
**3.125**	0.5	0.5	1
**0**	0.5	0.75	0.5

## Discussion

The increasing prevalence of MDR Gram-negative bacteria is a threat to global health and requires the development of new agents to treat infections caused by these organisms [1, 3, 18]. Although novel antimicrobial therapies are needed, drug development requires an immense amount of capital and time which delays their introduction into the clinic. Alongside the slow progression of drug discovery and development, a reassessment of ‘retired’ antibiotics and the inquiry into effective combinations with potential adjuvants are needed to prolong and enhance the utility of current antibiotics [20].

The purpose of this study was to investigate whether the antibacterial properties of sphingosine could enhance the activity of currently prescribed antibiotics against *P. aeruginosa*. Our initial screen of the effect of sphingosine versus a panel of antibiotics revealed that sphingosine increased the antibacterial activity of polymyxin B and colistin. We observed that polymyxin B and colistin synergize with sphingosine at multiple concentrations. The greatest degree of synergy with polymyxin B or colistin occurred with the addition of 50 µM sphingosine, resulting in an FICI of 0.263 and 0.35, respectively. These results were reflected in the enhanced bactericidal effects of polymyxin B when combined with sphingosine, best illustrated by the addition of 50 µM sphingosine, which reduced polymyxin B MBC from 0.5 to 0.063 µg ml^−1^. The other sphingoid bases, sphinganine and phytosphingosine, also enhanced polymyxin B activity. The potent antimicrobial activity of phytosphingosine was surprising as we previously reported that phytosphingosine does not affect the growth of *P. aeruginosa* unless the *sphBCD* operon was deleted [40]. Compared to wild-type *P. aeruginosa*, *sphBCD* deletion mutants display reduced growth in sphingosine, sphinganine and phytosphingosine with IC_50_ values of 84, 63 and 40 µM, respectively. This discrepancy is likely due to the differences in initial concentration of bacteria. In this study we started *P. aeruginosa* at an OD_600_ of 0.001 (corresponding to 1×10^6^ c.f.u. ml^−1^) [42], whereas previously we began bacterial cultures at an OD_600_ of 0.05 (5×10^7^ c.f.u. ml) [40]. Altered antimicrobial activity of sphingosine and phytosphingosine due to different concentrations of bacteria and sphingoid base has been reported previously [27, 28, 32]. In addition to *P. aeruginosa*, sphingosine enhanced the bacteriostatic and bactericidal activities of polymyxin B against two strains of *K. pneumoniae*, MGH78578 and KPPR1. The enhanced bacteriostatic effects were reflected in bacterial killing, where the addition of 200 µM sphingosine drastically reduced the concentration of polymyxin B needed to kill either strain of *K. pneumoniae*. Interestingly, antagonism between sphingosine and polymyxin B was observed when KPPR1 was exposed to 3.125 or 6.25 µM sphingosine, and these results were observed in all checkerboard assays and MBC assays.

To our knowledge, this is the first report of antibiotic synergy between polymyxins and sphingosine affecting the growth and survival of *P. aeruginosa*. Polymyxins, which have been clinically available for ~60 years, have been the focus of numerous studies, investigating their potential for combinatorial antibiotic therapies [52–59]. Additionally, the antimicrobial activity of sphingosines has been reported over the last three decades [9, 26–37, 40, 60–63]. Similar studies have been conducted using a sphingosine-1-phosphate analogue, fingolimod (FTY720), and demonstrated that fingolimod has antibacterial activity against Gram-positive bacteria [64]. Recently, a study conducted by Geng *et al*. [59] exhibited antibiotic synergy between fingolimod and colistin against *K. pneumoniae* which inhibited growth of *K. pneumoniae in vitro* and reduced mouse mortality in a murine pneumonia model. The authors hypothesized that the enhanced activity was due to the disruption of bacterial membranes by the combination and inhibition of membrane repair machinery by fingolimod, resulting in a loss of membrane integrity and the leakage of ATP [59]. A similar mechanism has been proposed for the antibacterial activity of sphingosines, where sphingosine induces continuous degradation of cardiolipin, resulting in membrane destabilization while exacerbating repair mechanisms, ultimately leading to death [31, 32]. It is generally understood that polymyxins disrupt outer membranes by electrostatically interacting with LPS and displacing the divalent cations (Mg^2+^ and Ca^2+^) for the insertion of its N-terminal fatty acyl group and hydrophobic amino acid side chains into the outer membrane fatty acyl layer [65, 66]. However, there are significant gaps in knowledge of the exact mechanism of action of polymyxins and the role antimicrobial adjuvants play in that mechanism. When paired with sphingosine, the combined interactions of sphingosine with cardiolipin and polymyxins with LPS may exacerbate membrane repair, leading to a rapid loss of membrane integrity. Alternatively, sphingosine may alter outer membrane fluidity to improve polymyxin entry. However, the inability of sphingosine to render *P. aeruginosa* susceptible to vancomycin or substantially reduce the efficacy of aminoglycoside antibiotics (Table 1**,** Figs S1, S2 and S4) suggests that sphingosine alone is unable to fully permeabilize the outer or inner membranes, respectively [57, 58]. Therefore, more work is needed to determine the mechanism of sphingosine and polymyxin synergy and convert clinical applications. Of particular interest in the future would be determining whether the different LPS modifications that drive most colistin resistance in both *K. pneumoniae* and *P. aeruginosa* prevent or permit the synergistic interaction between colistin and sphingosine.

Compared to polymyxins alone, the combination of polymyxin and sphingosine permits using significantly lower concentrations of polymyxins to kill Gram-negative bacteria, potentially mitigating adverse effects from polymyxin treatment, which has limited their clinical use. In the clinic, polymyxins are delivered intravenously to treat bacteraemia or by nebulized inhalation for the treatment of bacterial-induced pneumonia, the latter is associated with increased efficacy and reduced systemic adverse effects [67]. Inhalation of nebulized sphingosine has been demonstrated as safe and effective at reducing bacterial loads in porcine pneumonia models ex vivo and *in vivo* [60, 62, 63]. Therefore, inhalation of a sphingosine–polymyxin combination may prove to be an effective treatment for bacterial pneumonia. Alternatively, topical application of sphingosine and polymyxins could be used to treat infections in superficial cuts and lacerations, similar to common over-the-counter triple antibiotic ointments, which contain polymyxin B in combination with neomycin and bacitracin. Our data shows that sphingosine and polymyxins synergize to inhibit the growth of *P. aeruginosa* and *K. pneumoniae*; however, a caveat is that our experiments were conducted using a modified MOPS minimal media rather than the CLSI-recommended MHB. In addition to a defined Mg^2+^ and Ca^2+^ concentration, which would need to be adjusted in MHB [68], minimal media likely better reflects the lower growth rates of bacteria, including *P. aeruginosa*, during infection [69–74]. We have reported previously that the carbon source in minimal media impacts the effect of sphingosine on *P. aeruginosa* growth, where carbon sources enabling faster growth proportionally limit sphingosine’s growth-limiting effects [40]. However, when considering future applications of sphingosine–polymyxin combinations, their effectiveness should be tested in host-mimetic media that simulates the infectious environments of the skin, lung, eye and ear [75–77]. Another observation to note is that there is sphingosine present at epithelial surfaces and circulation. If additional sphingosine were to be added, there is the potential that total sphingosine would fall into the range that demonstrates antagonism in our data (Figs 4 and 7). Thus, it would be important to manage the colistin pharmacodynamics to avoid this possibility.

Overall, this study highlights the potential of sphingosine as an antimicrobial adjuvant for polymyxins against Gram-negative bacteria. The addition of sphingosine dramatically increased the bacteriostatic and bactericidal activities of colistin and polymyxin B against *P. aeruginosa* and *K. pneumoniae*. Although additional studies are needed to determine the combined mechanism of action and the efficacy of the combination in host-mimetic conditions, this pairing of polymyxins and sphingosine represents a prospect for future therapy.

## Supplementary material

10.1099/mic.0.001743 Fig. S1.

## Abbreviations

CF: cystic fibrosis
FIC: fractional inhibitory concentration
FICI: fractional inhibitory concentration index
MBC: minimum bactericidal concentration
MDR: multidrug resistant
MHB: Mueller–Hinton broth
