# Supplementary material for: Sphingosine synergizes with polymyxin antibiotics against Pseudomonas aeruginosa and Klebsiella pneumoniae
Source: Microbiology (Reading). 2026 Aug 4;172(8):001743. doi: 10.1099/mic.0.001743 (PMC13440347; doi:10.1099/mic.0.001743)
Supplement: Supplementary file 1 [file mic-172-01743-s001.pdf]

Supplemental Figures

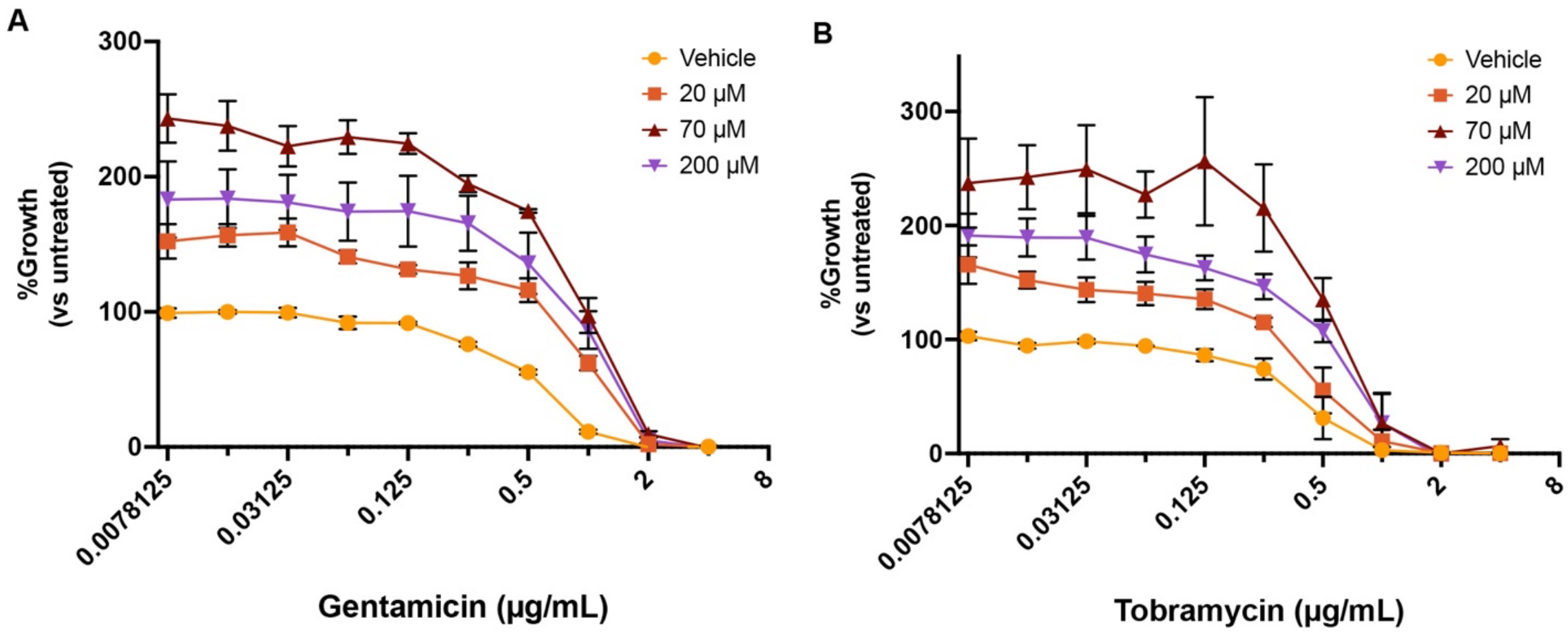

Supplemental Figure 1. Dose effect curves of PA14 in increasing concentrations of gentamicin **(A)** or tobramycin **(B)** alone or with 20 µM, 70 µM, and 200 µM sphingosine. Data points denote means summarizing three independent experiments and error bars represent the standard error of mean.

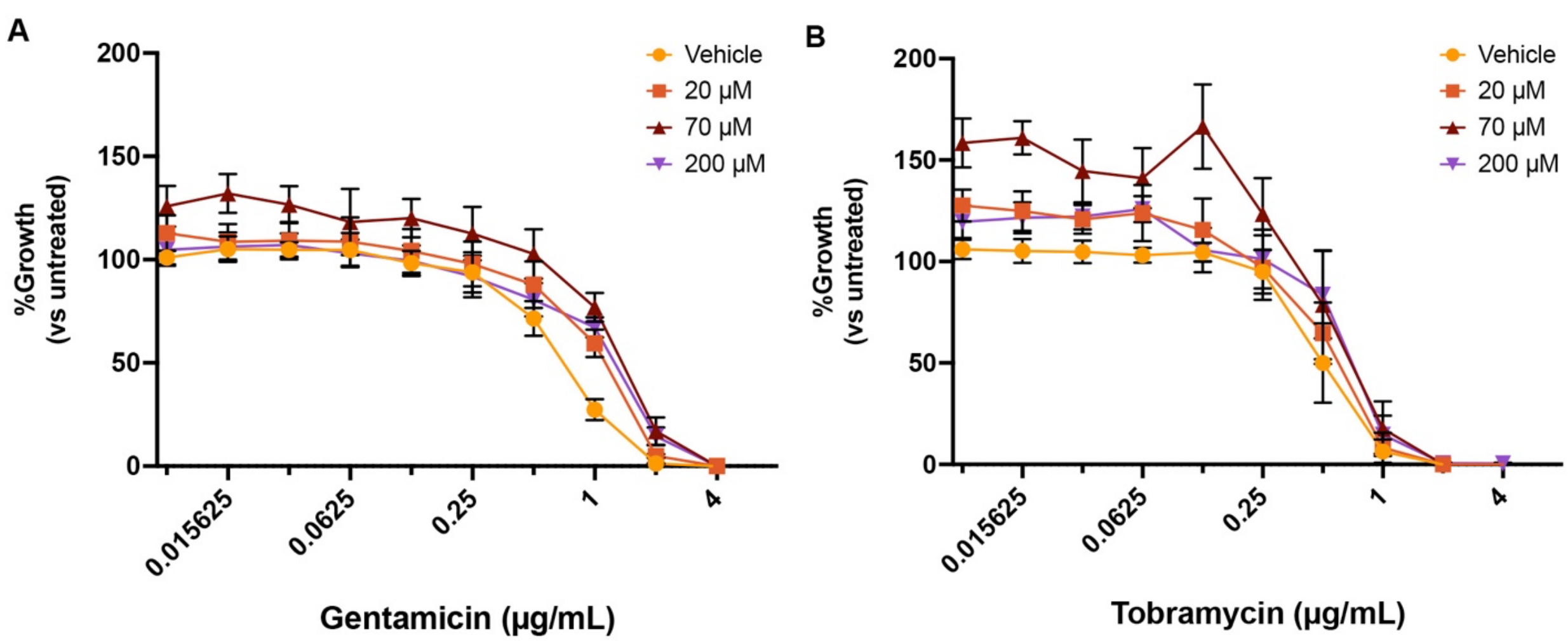

Supplemental Figure 2. Dose effect curves of PAO1 in increasing concentrations of gentamicin **(A)** or tobramycin **(B)** alone or with 20 µM, 70 µM, and 200 µM sphingosine. Data points denote means summarizing three independent experiments and error bars represent the standard error of mean.

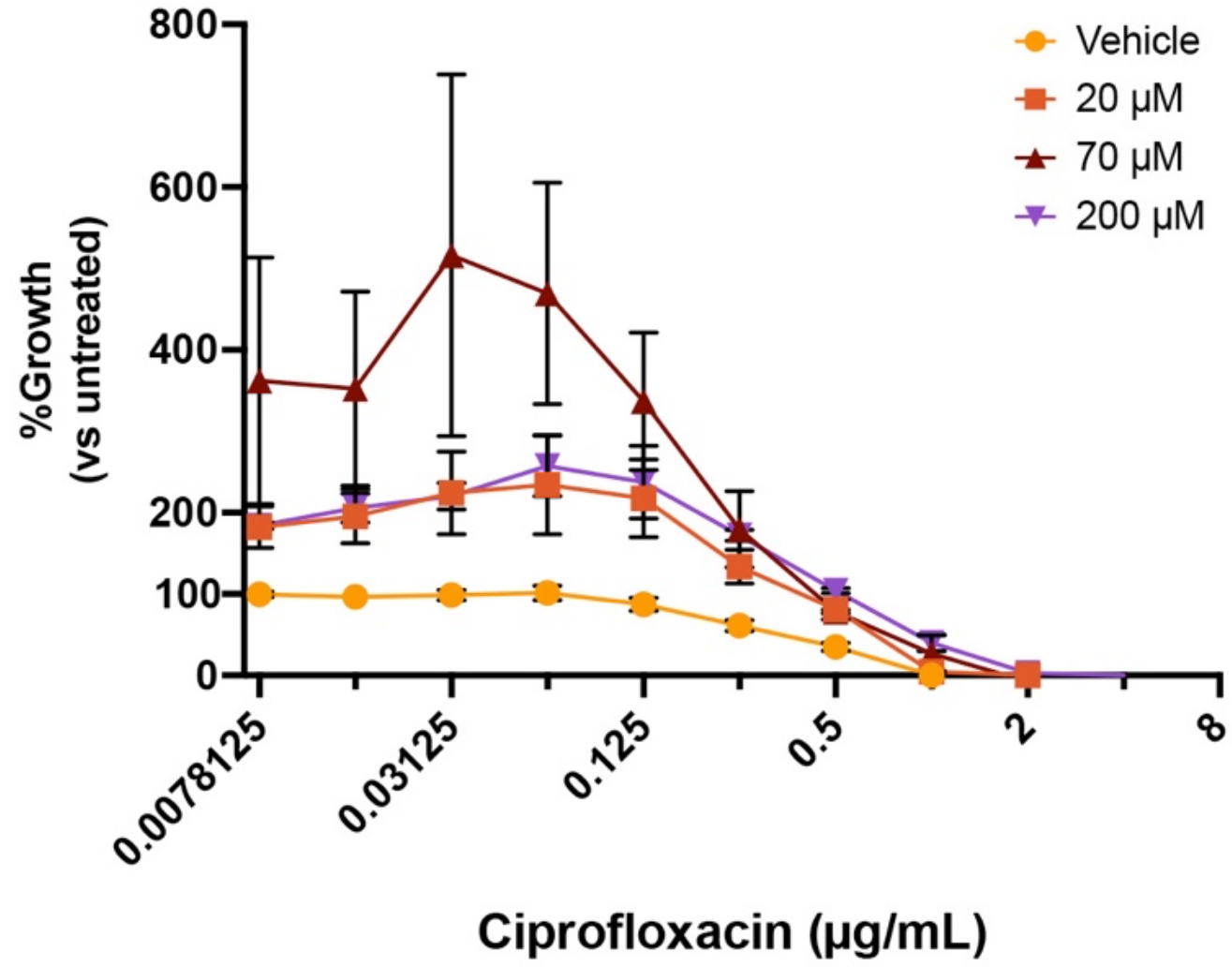

Supplemental Figure 3. Dose effect curves of PA14 in increasing concentrations of ciprofloxacin alone or with 20 µM, 70 µM, and 200 µM sphingosine. Data points denote means summarizing three independent experiments and error bars represent the standard error of mean.

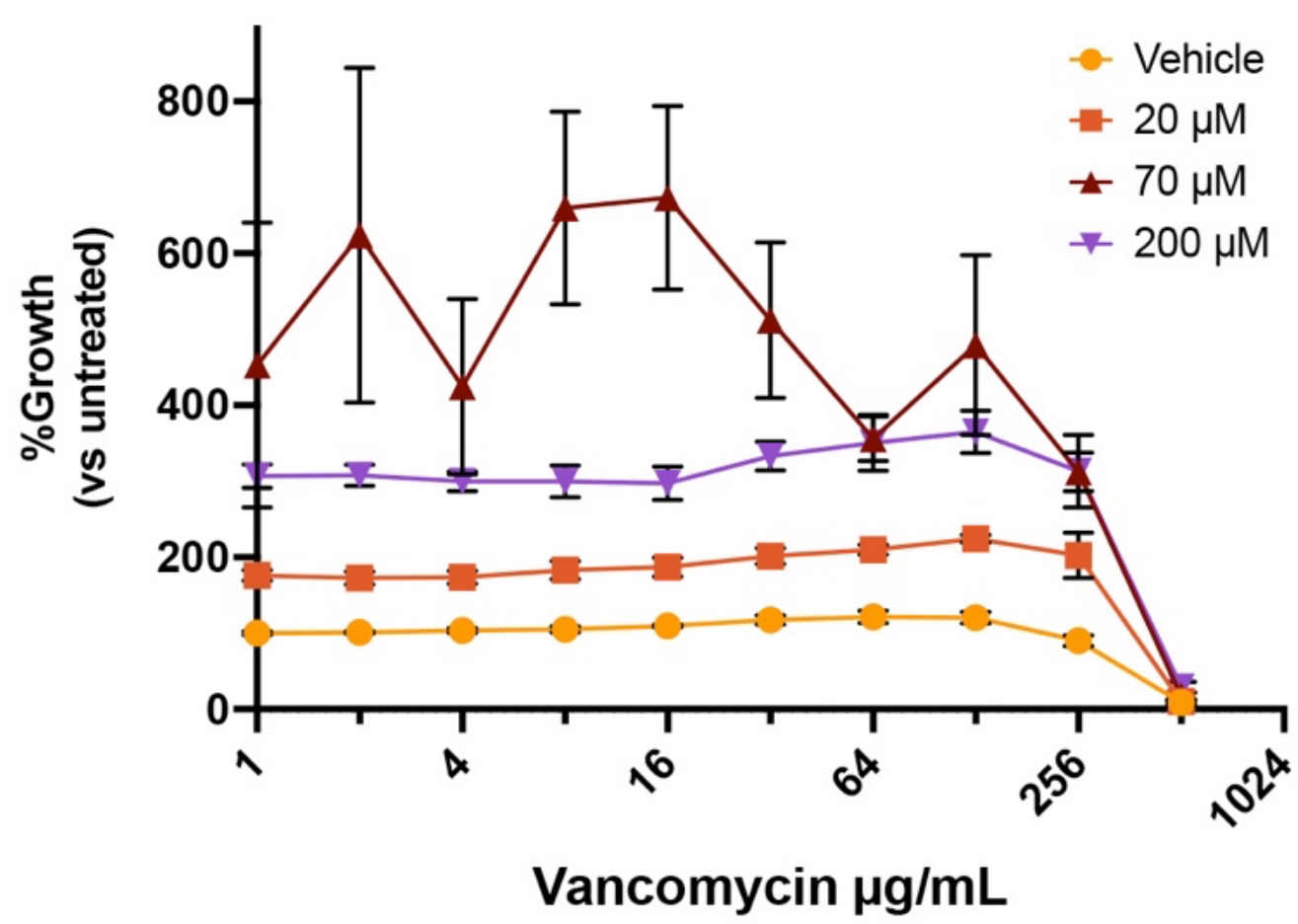

Supplemental Figure 4. Dose effect curves of PA14 in increasing concentrations of vancomycin alone or with 20 µM, 70 µM, and 200 µM sphingosine. Data points denote means summarizing three independent experiments and error bars represent the standard error of mean.

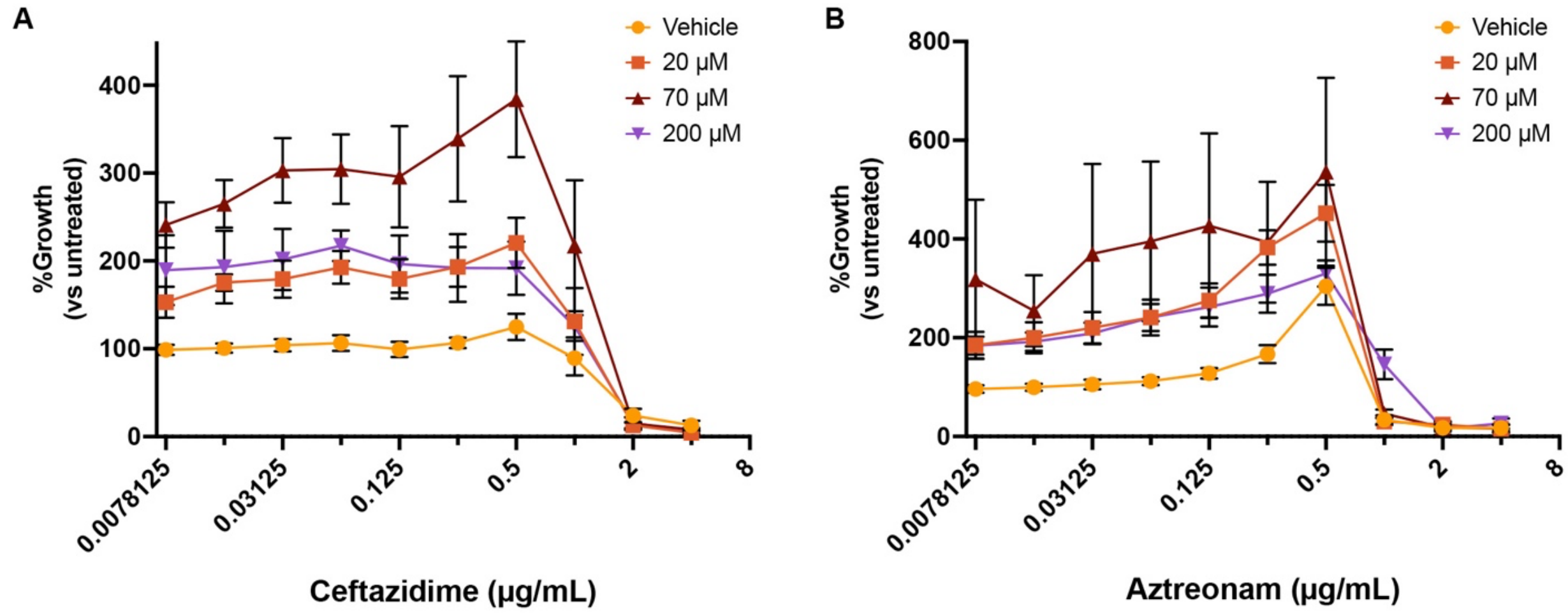

Supplemental Figure 5. Dose effect curves of PA14 in increasing concentrations of ceftazidime **(A)** or aztreonam **(B)** alone or with 20 µM, 70 µM, and 200 µM sphingosine. Data points denote means summarizing three independent experiments and error bars represent the standard error of mean.
